# One Health in the consciousness of veterinary students from the perspective of knowledge of antibiotic therapy and antimicrobial resistance: a multi-centre study

**DOI:** 10.3389/fpubh.2023.1165035

**Published:** 2023-05-24

**Authors:** Tomasz Sobierajski, Monika Wanke-Rytt, Wioleta Chajecka-Wierzchowska, Marcin Śmiałek, Waleria Hryniewicz

**Affiliations:** ^1^Faculty of Applied Social Sciences and Resocialization, The Center of Sociomedical Research, Warsaw University, Warsaw, Poland; ^2^Department of Pediatrics With Clinical Assessment Unit, Medical University of Warsaw, Warsaw, Poland; ^3^Department of Industrial and Food Microbiology, Faculty of Food Science, University of Warmia and Mazury in Olsztyn, Olsztyn, Poland; ^4^Department of Poultry Diseases, Faculty of Veterinary Medicine, University of Warmia and Mazury in Olsztyn, Olsztyn, Poland; ^5^Department of Epidemiology and Clinical Microbiology, National Medicines Institute, Warsaw, Poland

**Keywords:** AMR, carbapenems, animals, humans, public health, metabolic rift

## Abstract

One Health (OH) is one of the most essential global programs to rebalance the animal, human, and plant environments that depend on and affect each other. One element of the OH program is to draw attention to the phenomenon of antimicrobial resistance (AMR), which poses a very high risk to human and animal health. OH is not only a health-promoting project but also has an educational dimension. Therefore, a survey was conducted among 467 veterinary students studying at top academic centers in Poland to find out whether they had heard of OH and whether knowledge of OH influences their knowledge and attitudes related to AMR. The study indicated statistically significant relationships between familiarity with the OH program and the year of study. The higher the year of study, the more students heard about OH. It was also shown that students who had heard of OH were significantly more likely—compared to students who had not heard of OH—to agree that increasing AMR is influenced by the overuse of antibiotics in veterinary medicine (70.7 vs. 55%; *p* = 0.014) and the use of too low doses of antibiotics in animals (49.8 vs. 28.6%; *p* = 0.016). The higher the year of study, the higher the percentage of students who say that carbapenems as antibiotics of last resort should be reserved only for humans (70% of final-year students vs. 30.8% of first-year students; *p* < 0.001). The study's results indicate the effectiveness of education in fostering positive attitudes toward AMR and the impact of knowledge of the OH program on knowledge of antibiotic therapy in the spirit of OH.

## 1. Introduction

One Health (OH) encompasses a concept where the health of humans, animals (domestic and wild), plants, and the environment are closely linked and depend on each other (1). Interdisciplinary social and “metabolic rift” due to rapid, industrial, and urbanization development led to a reorganization of the global ecological balance (2, 3). The example of the COVID-19 pandemic showed how an OH approach that links people, animals, and plants could help control the spread of infectious diseases. By working together, OH will respond to health crises more quickly and manage them more efficiently to ensure global health security for people, animals, and plants (1). One Health is an interdisciplinary effort, implemented at different levels, to achieve optimal health for people, animals, and the environment in which people and animals live (4).

In an era of anthropocentrism, humans have the most significant responsibility for the health and security of the planet. For this reason, OH aims to familiarize people with the benefits of coordinating health activities across different sectors of human life and human activity at different levels—from local to regional to international to global.

The experience of recent decades with epidemics and global threats from the spread of zoonotic viruses (Ebola, Zika, SARS, H5N1, H7N9, MERS-CoV, and SARS CoV-2) has clearly shown how interconnected human and animal ecosystems are and the need for OH education (5). The interdependence of ecosystems, including animals and humans, has long been recognized. However, it was not until the second half of the 20th century that veterinary epidemiologist Calvin Schwabe clarified it and referred to it as “One Medicine” (6). His concept was one of the main elements that, by the end of the first decade of the 21st century, resulted in the One Health concept (7), which is being developed and promoted by major global organizations, including WHO, UNICEF, and World Bank (1, 8, 9).

One of the most crucial elements of the cross-sectoral and interdisciplinary OH effort is the prevention of antimicrobial resistance (AMR) by limiting antibiotic overuse, which is one of the main factors contributing to the development of AMR. While total antibiotic use in the European Union/European Economic Area (EA/EEA) area has declined by 23% in humans and 43% in animals (livestock) between 2011 and 2020, AMR in bacteria from humans living in the EU/EEA region has increased over the past decade (10). For several years, consumption in this regard has been higher in humans than in livestock, but at the same time, resistance to commonly used antibiotics in bacteria from livestock remains high (10). Companion animals have also been described as potential reservoirs of AMR, but data are still scarce. For example, one study evaluating the use of antibiotics in companion pets in three European countries found that as many as 19% of animals had received at least one antimicrobial treatment 6 months before a fecal sample was collected from the animals. Interestingly, 27% of the samples showed the presence of antibiotic-resistant *E. coli* isolates, of which as many as 13% were MDR (11). The use of antibiotics in pets was evaluated in a study by Bucland et al., which found that 25% of dogs and 21% of cats seen at veterinary practices participating in VetCompass in the UK received at least one antibiotic over 2 years (2012–2014) and 42% of these animals were given repeated antibiotics (12). From a One-Health perspective, companion animals might be a source of transmission of resistance genes and/or resistant bacteria to humans.

The experience of antibiotic reduction in veterinary medicine may serve as a model for reducing antibiotic consumption in humans. For this reason, awareness of the OH program's goals among physicians, veterinarians, and medical and veterinary students, and the reduction of antibiotic use in animals and humans, is crucial for global public health (10).

Researchers point out that the education of veterinary students should be focused on issues related to the assumptions of the OH concept and the role that veterinary graduates must play not only in ensuring global food but also in viewing the control of infectious diseases in livestock as going beyond veterinary medicine and relating to human health (13). For this reason, it is essential to look at the attitudes of veterinary students toward the One Health approach.

The results described in this article are part of a larger study. In early 2022, we published an article based on some survey results—mainly those related to students' knowledge and attitudes about antibiotic therapy (14).

In this article, we analyzed the students' awareness/lack of awareness of OH and focused on the role of carbapenems as antibiotics of last resort. Our goal was to examine whether students are aware of the approach and if and how awareness of the One Health approach affects their stated attitudes toward antibiotic use in animals.

For this study, based on the authors' previous knowledge and analysis of other sources in the literature, we adopted the following research hypotheses:

*H1. The higher the year of study, the greater the number of veterinary students aware of the One Health program*.
*H2. Students' declaration of awareness of the One Health assumptions has an impact on raising their awareness of the risks of antimicrobial resistance*.

## 2. Materials and methods

### 2.1. Study design and population

The survey was conducted in Poland's leading academic centers, training veterinary students: Warsaw University of Life Sciences, the University of Life Sciences in Lublin, the University of Warmia and Mazury in Olsztyn, and the University of Environmental and Life Sciences in Wrocław. The population of students in all years of veterinary medicine studying at these four universities was 3800. A total of 467 students participated in the study, which is a substantial quantitative sample for the entire population. Considering the size of this sample and the number of veterinary medicine students in Poland (*N* = 3,800), the maximum error was 3% for a confidence level of 95% and a fraction size of 0.5. The survey was conducted between May and June 2021.

### 2.2. The questionnaire and data collection

The interdisciplinary team of the article's authors prepared the questionnaire with the latest principles of qualitative survey preparation and implementation. The questionnaire consisted of three parts. The first part included questions about students' general knowledge of antibiotics, experiences with antibiotic therapy, assessment of their knowledge and that of others about antibiotics, and the need for education about antibiotics, antibiotic therapy, and AMR. The second part included questions testing knowledge of the causes that can cause or increase AMR and awareness of the global phenomenon of AMR. The third part included questions on microbiological testing, as well as recommendations on the use of antibiotics as a last resort.

The questionnaire was distributed at each academic center by specially prepared persons responsible for acquainting the respondents with the topic of the study and its purpose. Prior to the implementation of the survey proper, a pilot study was conducted to validate and verify the tool in terms of its structure and purpose.

### 2.3. Statistical analysis

All statistical analyses were performed in IBM SPSS Statistics 27.0.1.0. For all analyses, a *P*-level of < 0.05 was considered statistically significant. Cross-tabulations and chi-squared tests evaluated selected factors.

### 2.4. Ethical considerations

Study participants were informed that the study was anonymous and confidential, and results were collected and analyzed collectively to prevent any possibility of identifying the study participant. All students gave informed consent to participate in the study. The study received a favorable decision from the University of Warmia and Mazury in Olsztyn (Decision No. 15/2021).

## 3. Results

Two-thirds of the surveyed veterinary students (63.8%, *n* = 298) declared that they had not heard of the OH approach. The higher the year of study and the larger the class of locality, the more often the students had heard of OH (Table 1).

**Table 1:** Sociodemographic characteristics of respondents (by OH knowledge) (*N* = 467).

	**I have heard about OH**	**I have not heard about OH**	***p*-value**
**Gender**			
Female	132 (36.9)	226 (63.1)	0.753
Male	34 (33.3)	68 (66.7)	
Other	3 (42.9)	4 (57.1)	
**Year of study**			
I	10 (12.8)	68 (87.2)	< 0.001
II	24 (25.5)	70 (74.5)	
III	38 (42.2)	52 (57.8)	
IV	47 (44.3)	59 (55.7)	
V	37 (53.6)	32 (46.4)	
VI	13 (43.3)	17 (56.7)	
**University**			
Lublin	36 (45.8)	45 (54.2)	< 0.001
Olsztyn	50 (25.3)	148 (74.7)	
Warszawa	45 (69.2)	20 (30.8)	
Wrocaw	36 (29.8)	85 (70.2)	
**Place of origin**			
Village with a farm	22 (36.7)	38 (63.3)	0.011
Village without a farm	24 (26.1)	68 (73.9)	
City < 50 k	30 (28.6)	75 (71.4)	
City 50–200 k	38 (42.2)	52 (57.8)	
City > 200 k	55 (45.8)	65 (54.2)	

Students who declared that they had heard of OH gave themselves a slightly better rating regarding their knowledge of antibiotics (*p* = 0.073). Their knowledge of antibiotics was rated as good or very good by nearly four in 10 respondents (38.5%) who said they had heard of OH and nearly three in 10 respondents (27.9%) who said they had not heard of OH.

Apart from the impact of misuse of antibiotics in veterinary medicine (*p* = 0.014) and using too low doses of antibiotics in animals on AMR (*p* = 0.016), there were no significant differences in knowledge regarding antibiotic use and the impact on AMR (Table 2).

**Table 2:** To what extent do you agree that the following attitudes/behaviors, in your opinion, have an impact on the growth of AMR? (by OH knowledge) (*N* = 467).

	**Totally disagree**	**Disagree**	**Rather disagree**	**Rather agree**	**Agree**	**Totally agree**	***p*-value**
**Over-prescription antibiotic by physicians**							
I have heard about OH	2 (1.2)	6 (3.6)	22 (13.0)	24 (14.2)	44 (26.0)	71 (42.0)	0.823
I have not heard about OH	7 (2.3)	14 (4.7)	46 (15.4)	44 (14.8)	76 (25.5)	111 (37.2)	
**Misuse of antibiotics in veterinary medicine**							
I have heard about OH	6 (3.6)	12 (7.1)	30 (17.8)	31 (18.3)	53 (31.4)	37 (21.0)	0.014
I have not heard about OH	14 (4.7)	47 (15.8)	73 (24.5)	44 (14.8)	66 (22.1)	54 (18.1)	
**Low awareness of the risks of antibiotic resistance phenomenon**							
I have heard about OH	2 (1.2)	3 (1.8)	14 (8.3)	11 (6.5)	49 (29.0)	90 (53.3)	0.661
I have not heard about OH	3 (1.0)	10 (3.4)	30 (10.1)	29 (9.7)	81 (27.2)	145 (48.7)	
**Use of antibiotics in fattening livestock**							
I have heard about OH	10 (5.9)	12 (7.1)	27 (16.0)	37 (21.9)	39 (23.1)	44 (26.0)	0.450
I have not heard about OH	23 (7.7)	34 (11.4)	57 (19.1)	61 (20.5)	58 (19.5)	65 (21.8)	
**Excessive antibiotic therapy in animals**							
I have heard about OH	20 (11.8)	27 (16.0)	43 (25.4)	34 (20.1)	25 (14.8)	20 (11.8)	0.211
I have not heard about OH	25 (8.4)	39 (13.1)	93 (31.2)	80 (26.8)	36 (12.1)	25 (8.4)	
**Using too low doses of antibiotics in animals**							
I have heard about OH	16 (9.5)	28 (16.6)	41 (24.3)	29 (17.2)	29 (17.2)	26 (15.4)	0.016
I have not heard about OH	31 (10.4)	55 (18.5)	97 (32.6)	62 (10.8)	30 (10.1)	23 (7.7)	
**Low level of hygiene in breeding**							
I have heard about OH	6 (3.6)	11 (6.5)	34 (20.1)	26 (15.4)	36 (21.3)	56 (33.1)	0.134
I have not heard about OH	8 (2.7)	43 (14.4)	51 (17.1)	45 (15.1)	71 (23.8)	80 (26.8)	

Two-thirds of students (65.2%, *n* = 305) said that direct contact with companion animals contributes little or nothing to the likelihood of a population resistant microorganisms in humans: 23.3% (*n* = 109) said it “has no impact at all,” 24.6% (*n* = 115) said it contributes to a “negligible extent,” and 17.3% (*n* = 81) said it contributes to a “small extent.” One in seven (16.3%, *n* = 76) students said it has any influence, and 18.4% (*n* = 86) of students had no opinion on the subject. Students who had not heard of the One Health approach were slightly more likely to declare that direct contact with companion animals makes little or no contribution to the likelihood of a population of resistant microorganisms in humans than students who had heard of the approach.

Six out of 10 students (58.2%, *n* = 272) believed that direct contact with farm animals does not contribute or contributes little to the likelihood of resistant microbial populations in humans, with 18.6% of students (*n* = 87) believing that it “has no effect at all,” 21.4% of students (*n* = 100) that it contributes to a “negligible degree,” and 18.2% of students (*n* = 85) that it contributes to a “small degree.” One in five (20.9%, *n* = 98) students said it has any influence, and 20.8% (*n* = 97) of students had no opinion on the subject. Students who had not heard of the One Health approach were more likely to declare that direct contact with farm animals contributes little or nothing to the likelihood of a population of resistant microorganisms in humans than students who had heard of the approach.

One-third of the students (36.4%, *n* = 150) felt that the consumption of animal products (e.g., eggs, milk, and meat) does not contribute or contributes little to the likelihood of resistant microbial populations in humans, with 8.6% of students (*n* = 40) felt that it “has no effect at all,” 14.1% of students (*n* = 66) that it contributes to a “negligible degree,” and 13.7% of students (*n* = 64) felt that it contributes to a “small degree.” One in two (47.8%, *n* = 287) students thought it had any impact, and 15.8% (*n* = 74) of students had no opinion on the subject. Students who had not heard of the One Health approach were more likely to declare that the consumption of animal products (e.g., eggs, milk, and meat) contributes little or nothing to the likelihood of a population of resistant microorganisms in humans than students who had heard of the approach.

Three-fourths of the students (76.0%, *n* = 240) believed that the consumption of plant-based products does not contribute or contributes little to the likelihood of resistant microbial populations in humans, with 33.2% of students (*n* = 155) believing that it “has no impact at all,” 27.2% of students (*n* = 127) that it contributes to a “negligible extent,” and 15.6% of students (*n* = 73) that it contributes to a “small extent.” One in 10 (10.7%, *n* = 50) students thought it had any impact, and 13.3% (*n* = 64) had no opinion on the subject. Students who had not heard of the One Health approach were slightly more likely to declare that consuming plant-based products contributes little or nothing to the likelihood of human-resistant microbial populations than students who had heard of the approach.

Two-thirds (63.3%, *n* = 296) of the students disagreed that selected antibiotics of last resort (e.g., carbapenems) should be reserved for use only in humans (18.4%, *n* = 86 for the “strongly disagree” response; 18.6%, *n* = 87 for the “disagree” response; 26.3%, *n* = 123 for the “rather disagree” response). One in seven students (15.2%, *n* = 71) “strongly agreed” with this opinion.

By the year of study, students in the fifth and sixth years were more likely to “strongly agree” with it than students in lower years (Table 3).

**Table 3:** In your opinion, should selected antibiotics of last resort (e.g., carbapenems) be restricted for use only in humans? (by year of study) (*N* = 467).

	**Totally disagree**	**Disagree**	**Rather disagree**	**Rather agree**	**Agree**	**Totally agree**	***p*-value**
I	14 (17.9)	12 (15.4)	28 (35.9)	12 (15.4)	10 (12.8)	2 (2.6)	< 0.001
II	21 (22.3)	21 (22.3)	38 (40.4)	9 (9.6)	2 (2.1)	3 (3.2)	
III	14 (15.6)	23 (25.6)	22 (24.4)	9 (10.0)	4 (4.4)	18 (20.0)	
IV	24 (22.6)	19 (17.9)	19 (17.9)	14 (13.2)	13 (12.3)	17 (16.0)	
V	11 (15.9)	8 (11.6)	13 (18.8)	11 (15.9)	5 (7.2)	21 (30.4)	
VI	2 (6.7)	4 (13.3)	3 (10.0)	5 (16.7)	6 (20.0)	10 (33.3)	

**Figure 1:**
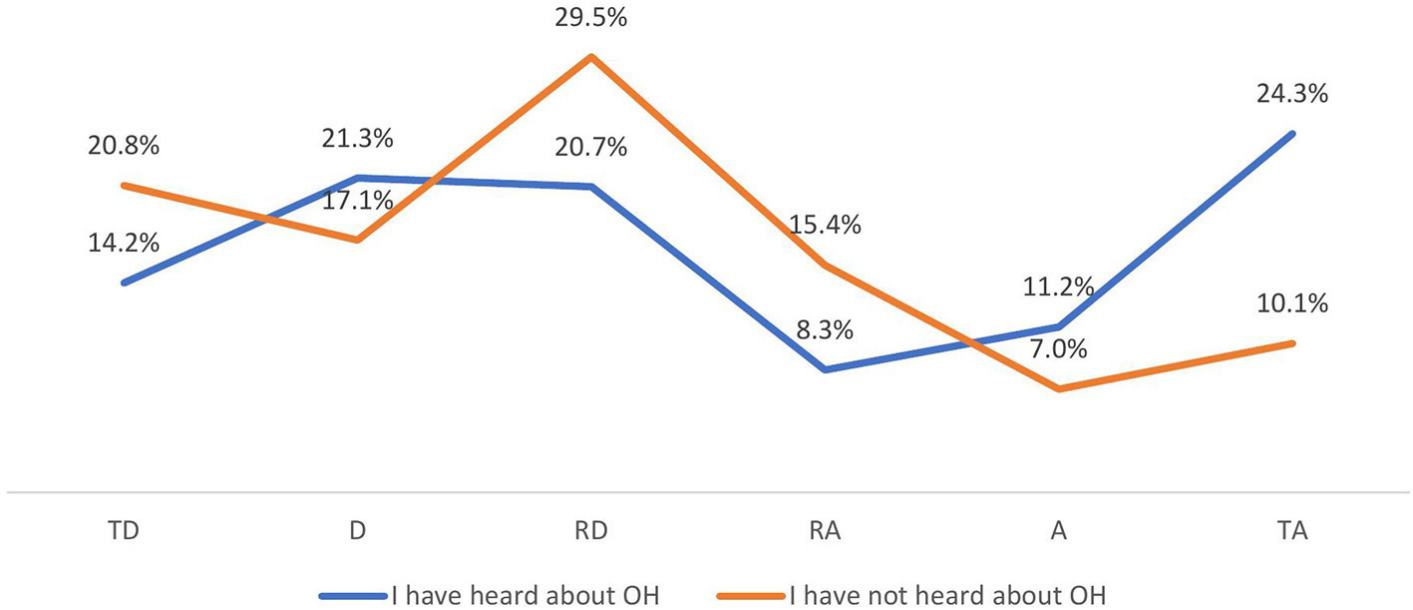
In your opinion, should selected antibiotics of last resort (e.g., carbapenems) be restricted for use only in humans? (by OH knowledge) (*N* = 467). TD, totally disagree; D, disagree; RD, rather disagree; RA, rather agree; A, agree; TA, totally agree.

Students who have heard of OH are significantly more likely to be convinced that carbapenems should be reserved for use only in humans than students who have not heard of OH (*p* < 0.001) (Figure 1).

**Figure 2:**
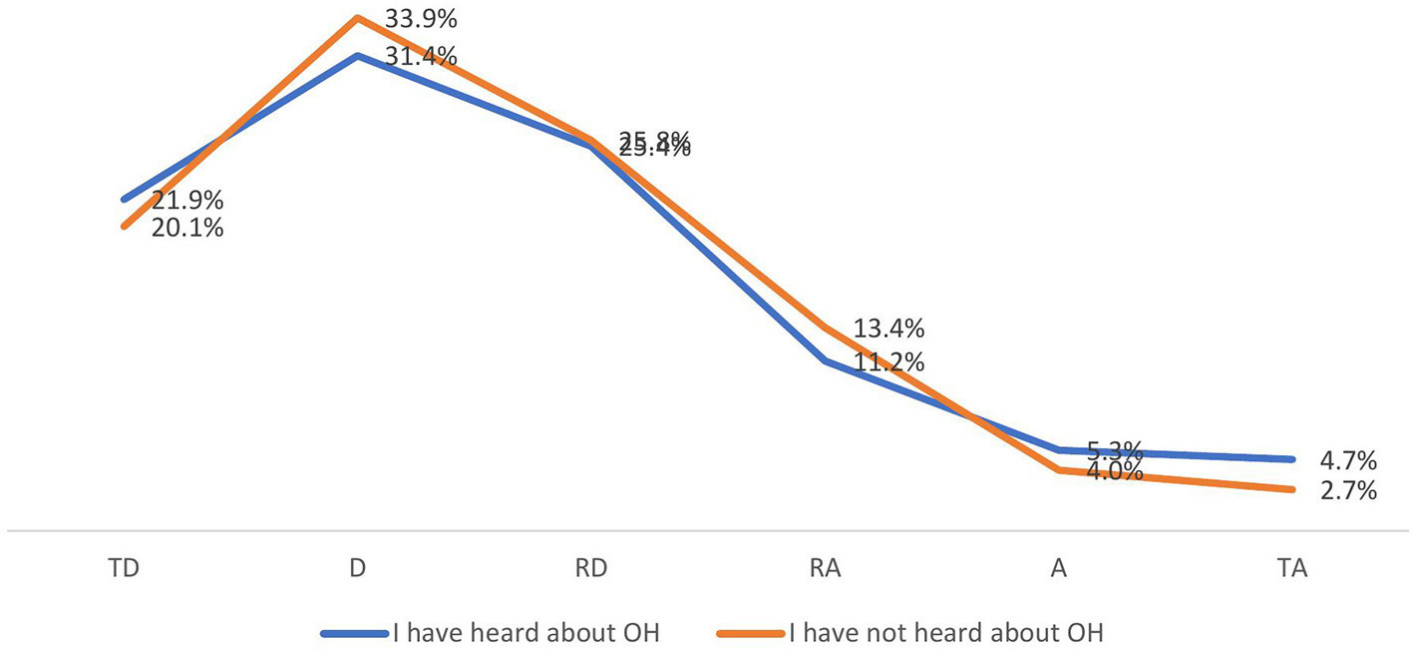
Do you think the public is adequately informed about such veterinary public health aspects as testing for Salmonella, testing meat for antibiotics, and official controls on compliance with grace periods for antibiotics used? (by OH knowledge) (*N* = 467). TD, totally disagree; D, disagree; RD, rather disagree; RA, rather agree; A, agree; TA, totally agree.

According to most students, the public in Poland is not adequately informed about such veterinary aspects of ensuring public health as, e.g., testing for Salmonella, testing meat for antibiotics, or official controls on adherence to withdrawal periods for antibiotics used (20.8%, *n* = 97 for “definitely not informed” responses; 33%, *N* = 154 for “not informed” responses; 25.7%, *n* = 120 for “rather not informed” responses (Figure 2).

Sixth-year students agreed to the highest degree that the public needs to be adequately informed (Table 4).

**Table 4:** Do you think the public is adequately informed about such veterinary public health aspects as testing for Salmonella, testing meat for antibiotics, and official controls on compliance with grace periods for antibiotics used? (by year of study) (*N* = 467).

	**Totally disagree**	**Disagree**	**Rather disagree**	**Rather agree**	**Agree**	**Totally agree**	***p*-value**
I	2 (2.6)	22 (28.2)	30 (38.5)	20 (25.6)	1 (1.3)	3 (3.8)	< 0.001
II	17 (18.1)	37 (39.4)	25 (26.6)	9 (9.6)	6 (6.4)	0 (0)	
III	21 (23.3)	34 (37.8)	22 (24.4)	7 (7.8)	4 (4.4)	2 (2.2)	
IV	24 (22.6)	33 (31.1)	26 (24.5)	7 (6.6)	9 (8.5)	7 (6.6)	
V	20 (29.0)	22 (31.9)	12 (17.4)	12 (17.4)	1 (1.4)	2 (2.9)	
VI	13 (43.3)	6 (20.0)	5 (16.7)	4 (13.3)	0 (0)	2 (6.7)	

## 4. Discussion

The purpose of our study was to see if and how awareness of One Health understood by the veterinary students translates into their attitudes toward antibiotic therapy in animals and humans and their awareness of the dangers associated with AMR. Our study showed a statistically significant relationship between the year of study, place of residence, and awareness of OH, which confirms hypothesis H1. *The higher the year of study, the greater the number of veterinary students aware of the One Health program*. A study by Subedi et al. that verified veterinary students' knowledge of the OH program also indicated a significant relationship between the academic studies progression (level of study) and acquired knowledge of OH (15). The authors also point out that veterinary students should be even more involved in OH activities, particularly earlier in their studies (15). In some developed countries, such as the US, One Health knowledge was already recognized over a decade ago as a core competency for all graduating veterinarians (5, 16).

For this reason, many universities training veterinarians have introduced elements of OH into their curricula. This resulted in a higher level of familiarity with OH among US students, as indicated by a study by Wong and Kogan, in which as many as three-quarters of veterinary students surveyed (74.2%) were aware of the OH program (17). In 2021, Iatridou et al. published an article assessing, based on a questionnaire survey, whether interdisciplinary OH topics were introduced into the curriculum at European veterinary universities (18). Data from 41 institutions showed that interdisciplinary education for students still needs to be improved and requires further reinforcement to make OH an integral part of the university curriculum. Thirteen of the 41 universities (31.71%) said they organize courses for undergraduate students, whereas 14 VEEs (34.15%) acknowledged that they need to organize interdisciplinary training, and 14 VEEs (34.15%) do not organize or plan to adopt any interdisciplinary teaching in future (18). In our study, one of a third of all the students were aware of the OH program, which may indicate that the information about OH during their veterinary studies and interships, was well presented. It is also interesting to juxtapose the results related to the awareness of the existence of the OH program with the place of residence. Students from rural areas whose parents owned a farm heard about OH significantly more often than those whose parents did not (*p* = 0.011). The different levels of knowledge of One Health depending on the background were also demonstrated by Wilkes et al. The authors proposed educational models to overcome this problem, as well as the use of the OH concept to promote the introduction of interdisciplinary teamwork in veterinary college classes (19).

Our study showed a statistically significant relationship between the knowledge of OH and awareness of antibiotic overuse in veterinary medicine, which confirms the hypothesis H2. *Students' declaration of awareness of the OH assumptions have an impact on raising their awareness of the risks of antimicrobial resistance*. The results regarding the influence of the use of inadequate doses of antibiotics in animal husbandry and the abuse of antibiotics in veterinary medicine on the awareness of the impact of these factors on the increase of AMR positively verified our hypothesis, in which we state that the higher the year of study, the greater the knowledge of students about the risks of using inappropriate antibiotic therapy and the impact of these actions on the increase of AMR.

The AMR phenomenon is very relevant to OH. Indeed, there is scientific evidence that the overuse of antibiotics in veterinary medicine has led to the emergence of resistant bacteria which may cause infections in humans (20, 21). Sikkema and Koopmans, who analyzed OH research and education activities in Western Europe, pointed out that OH is promoted mainly in this part of the world for AMR where awareness of its consequences is very high. It is primarily through governmental programs to combat AMR that One Health knowledge is introduced (22). The authors also point out that although OH appears in the health policy documents of many countries, there is still a need for cross-sectoral cooperation, and the main OH activities are initiated by the veterinary sector (22). This may explain Polish veterinary students' markedly better knowledge of AMR from this study compared to Polish medical students' knowledge of AMR (23). In a meta-analysis published in 2022, Velazquez-Meza et al. point out that most antibiotics are available for humans and animals, and AMR can be reduced if antibiotics are used only for treatment, rarely for prevention, but never as growth promoters (24) which were banned in the EU in 2006 but in the USA in 2017.

As many antibiotics are used in humans and animals, we asked students about their attitudes toward using carbapenems in veterinary medicine. In Poland, carbapenems use is limited to treating confirmed or suspected severe infections caused by multidrug-resistant organisms and is reserved for humans only and is not licensed for veterinary use (25, 26). In our study, we indicated a statistically significant relationship between the year of study and OH knowledge and belief in using carbapenems as an antibiotic of last resort in humans. Our survey results indicate that the knowledge of carbapenems increases from the third year of study, which is when pharmacology and clinical classes begin. In Poland, as in the rest of the European Union, carbapenems are reserved only for the treatment of infections in humans, and from a global point of view, this should not affect the development of resistance to this group of antibiotics. However, other antibiotics could be blamed for the emergence of resistance besides the overuse of carbapenems, especially fluoroquinolones and third-generation cephalosporins. Therefore, we should aim to decrease all antibiotic consumption.

However, the use of carbapenems in veterinary medicine is permitted in many countries. For example, at a US veterinary center, based at the University of Pennsylvania, between 2013 and 2018, carbapenems were prescribed empirically in more than half of the patients (27). The results of a medical analysis in one US veterinary hospital indicated that most carbapenems used in animals were prescribed in conjunction with culture and antibiogram, and carbapenem resistance was found in 3% of the bacterial isolates with carbapenem susceptibility tested (28). A cross-sectional Italian study of hospitalized and non-hospitalized pets found that the prevalence of carbapenem-resistant bacteria in pets was 11.4% in hospitalized animals and 1.0% in non-hospitalized animals, indicating the need for greater control of carbapenem prescribing in veterinary care in developed countries, including the European Union (29).

An interesting hypothesis was put forward by Lei et al., who claim that the co-occurrence in humans and animals (especially companion animals) of the same multidrug-resistant strains may be due not only to previous antibiotic therapy in animals (or humans) and interspecies transfer of resistance but also to the fact that domestic animals share the same environments and food with humans (30). Finally, it underscores how important it is to take an interdisciplinary look at the issue of AMR, which is precisely the premise of One Health (30).

The OH paradigm needs to be changed in many fields: cultural and educational in many specialties and at different levels of education. We agree with the conclusions made in the publication by Iatridou et al., in which the authors propose five recommendations to promote interdisciplinary education in veterinary curricula:

- The need to develop transdisciplinary One Health competencies in the curricula of various disciplines in the EU
- The need for an integrated strategy in universities (for undergraduate and postgraduate students) to encourage and support interdisciplinarity
- The need for a harmonized approach to academic education through accreditation
- The need for appropriate legislation to facilitate interdisciplinary training
- The need to encourage interdisciplinary research on the One Health phenomenon (18).

### 4.1. Study limitations

Our study had some limitations. First, the study included four veterinary schools in Poland. Although these are the largest academic centers in veterinary medicine and are in different regions of the country, it would be worthwhile to examine students from other, smaller centers in future studies. In our analysis, we relied mainly on students' questionnaires and did not directly check their knowledge of One Health.

## 5. Conclusion

One Health is one of the most important inter-environmental and interdisciplinary health programs, which refers to the global metabolic cycle in which humans, animals, and plants depend on each other. Our survey identified some gaps related to students' awareness of OH. At the same time, our study provides evidence that the knowledge of One Health and the phenomenon of correct antibiotic use in animals and humans increases with the year of study. However, there is still a substantial educational space to equip veterinary students with competencies related to OH knowledge and its practical application.

## Data availability statement

The raw data supporting the conclusions of this article will be made available by the authors, without undue reservation.

## Ethics statement

The studies involving human participants were reviewed and approved by University of Warmia and Mazury in Olsztyn (Decision No. 15/2021). Written informed consent for participation was not required for this study in accordance with the national legislation and the institutional requirements.

## Author contributions

TS, MW-R, WC-W, MŚ, and WH: conceptualization of the study. TS: questionnaire preparation, methodology, statistical analysis, and writing an original draft. WC-W, MŚ, and TS: data curations. MW-R, TS, and WH: editing of original draft, review and supervision. All authors contributed to the article and approved the submitted version.

## References

[1] World Health Organization One Health (2022). Available online at: https://www.who.int/health-topics/one-health#tab=tab_1 (accessed September 21, 2022).

[2] Moore JW. Environmental crises and the metabolic rift in world-historical perspective. Org Environ. (2000) 13:123–57. 10.1177/1086026600132001

[3] Foster JB. Marx's theory of metabolic rift: classical foundations for environmental sociology. Am J Sociol. (1999) 105:366–405. 10.1086/210315

[4] King LJ Anderson LR Blackmore CG Blackwell MJ Lautner EA Marcus LC. Executive summary of the AVMA One Health Initiative Task Force report. J Am Vet Med Assoc. (2008) 233:259–61. 10.2460/javma.233.2.259 18627228

[5] Mackenzie JS McKinnon M Jeggo M. One Health: from concept to practice. Confront Emerg Zoonoses. (2014) 2014:163–89. 10.1007/978-4-431-55120-1_8

[6] Schwabe CW. Veterinary Medicine and Human Health. Baltimore: Williams & Wilkins (1984). 680 p.

[7] Kaplan B. “*One Medicine-One Health”: And Historic Perspective*. (2022). Available online at: https://onehealthinitiative.com/wp-content/uploads/2022/08/One-Medicine-One-Health-An-Historic-Perspective-REVISED-SEPT1-2022-from-FEB1-2021.pdf (accessed September 21, 2022).

[8] UNESCO. Preventing the Next Pandemic: One Health Approach. (2022). Available online at: https://www.unesco.org/en/articles/preventing-next-pandemic-one-health-approach?TSPD_101_R0 (accessed November 2, 2022).

[9] World Bank Group. One Health: Operational Framework for Strengthening Human, Animal, and Environmental Public Health Systems at Their Interface. (2018). Available online at: https://documents1.worldbank.org/curated/en/703711517234402168/pdf/123023-REVISED-PUBLIC-World-Bank-One-Health-Framework-2018.pdf (accessed November 6, 2022).

[10] ECDC EFSA, EMA, OECD,. Antimicrobial Resistance in the EU/EEA. A One Health Response. (2022). Available online at: https://www.ecdc.europa.eu/sites/default/files/documents/antimicrobial-resistance-policy-brief-2022.pdf (accessed September 1, 2022).

[11] Joosten P Ceccarelli D Odent E Sarrazin S Graveland H Van Gompel L. Antimicrobial usage and resistance in companion animals: a cross-sectional study in three European countries. Antibiotics. (2020) 9:87. 10.3390/antibiotics9020087 32079072 PMC7175148

[12] Buckland EL O'Neill D Summers J Mateus A Church D Redmond L. Characterisation of antimicrobial usage in cats and dogs attending UK primary care companion animal veterinary practices. Vet Rec. (2016) 179:489. 10.1136/vr.103830 27543064

[13] Kelly AM Ferguson JD Galligan DT Salman M Osburn BI. One health, food security, and veterinary medicine. J Am Vet Med Assoc. (2013) 242:739–43. 10.2460/javma.242.6.739 23445280

[14] Sobierajski T Mazińska B Chajecka-Wierzchowska W Smiałek M Hryniewicz W. Antimicrobial and antibiotic resistance from the perspective of Polish veterinary students: an inter-university study. Antibiotics. (2022) 11:115. 10.3390/antibiotics11010115 35052992 PMC8772817

[15] Subedi D Gautam A Sapkota D Subedi S Sharma S Abdulkareem M et al. Knowledge and perception of veterinary students on One Health: a first nationwide multi-institutional survey in Nepal. Int J One Health. (2022) 8:34–42. 10.14202/IJOH.2022.34-42

[16] Gibbs SE Gibbs EP. The historical, present, and future role of veterinarians in One Health. Curr Top Microbiol Immunol. (2013) 365:31–47. 10.1007/82_2012_259 22911439 PMC7121980

[17] Wong D Kogan LR. Veterinary students' attitudes on One Health: implications for curriculum development at veterinary colleges. J Vet Med Educ. (2013) 40:58–62. 10.3138/jvme.0612.057R 23475413

[18] Iatridou D Bravo A Saunders J. One Health interdisciplinary collaboration in veterinary education establishments in Europe: mapping implementation and reflecting on promotion. J Vet Med Educ. (2021) 48:427–40. 10.3138/jvme-2020-0019 33657331

[19] Wilkes MS Conrad PA Winer JN. One Health-One education: medical and veterinary inter-professional training. J Vet Med Educ. (2019) 46:14–20. 10.3138/jvme.1116-171r 30418808

[20] Bager F Madsen M Christensen J Aarestrup FM. Avoparcin used as a growth promoter is associated with the occurrence of vancomycin-resistant *Enterococcus faecium* on Danish poultry and pig farms. Prev Vet Med. (1997) 31:95–112. 10.1016/S0167-5877(96)01119-1 9234429

[21] Dutil L Irwin R Finley R Ng LK Avery B Boerlin P et al. Ceftiofur resistance in *Salmonella enterica* serovar Heidelberg from chicken meat and humans, Canada. Emerg Infect Dis. (2010) 16:48–54. 10.3201/eid1601.090729 20031042 PMC2874360

[22] Sikkema R Koopmans M. One Health training and research activities in Western Europe. Infect Ecol Epidemiol. (2016) 6:33703. 10.3402/iee.v6.33703 27906121 PMC5131506

[23] Sobierajski T Mazińska B Wanke-Rytt M Hryniewicz W. Knowledge-based attitudes of medical students in antibiotic therapy and antibiotic resistance. A cross-sectional study. Int J Environ Res Public Health. (2021) 18:3930. 10.3390/ijerph18083930 33918039 PMC8068920

[24] Velazquez-Meza ME Galarde-López M Carrillo-Quiróz B Alpuche-Aranda CM. Antimicrobial resistance: One Health approach. Vet World. (2022) 15:743–749. 10.14202/vetworld.2022.743-749 35497962 PMC9047147

[25] Jessen LR Damborg P Spohr A Goericke-Pesch S Langhorn R Houser G. Antibiotic Use Guidelines for Companion Animal Practice. Companion Animal Group, Danish Veterinary Association (2018) (Polish edition). Available online at: https://www.ddd.dk/media/4440/poland-antybiotyki-u-zwierzat-towarzysz-a-cych.pdf (accessed November 2, 2022).

[26] Clinical guidelines for antimicrobial treatment in companion animal practice. Vet Rec. (2021) 189:313. 10.1002/vetr.1097 34677867

[27] Cole SD Perez-Bonilla D Hallowell A Redding LE. Carbapenem prescribing at a veterinary teaching hospital before an outbreak of carbapenem-resistant *Escherichia coli*. J Small Anim Pract. (2022) 63:442–6. 10.1111/jsap.13481 35262929

[28] Smith A Wayne AS Fellman CL Rosenbaum MH. Usage patterns of carbapenem antimicrobials in dogs and cats at a veterinary tertiary care hospital. J Vet Intern Med. (2019) 33:1677–85. 10.1111/jvim.15522 31119803 PMC6639476

[29] Gentilini F Turba ME Pasquali F Mion D Romagnoli N Zambon E et al. Hospitalized pets as a source of carbapenem-resistance. Front Microbiol. (2018) 9:2872. 10.3389/fmicb.2018.02872 30574124 PMC6291488

[30] Lei L Wang Y He J Cai C Liu Q Yang D et al. Prevalence and risk analysis of mobile colistin resistance and extended-spectrum β-lactamase genes carriage in pet dogs and their owners: a population-based cross-sectional study. Emerg Microbes Infect. (2021) 10:242–51. 10.1080/22221751.2021.1882884 33502946 PMC7889244

