# Supplementary material for: One Health in the consciousness of veterinary students from the perspective of knowledge of antibiotic therapy and antimicrobial resistance: a multi-centre study
Source: Front Public Health. 2023 May 24;11:1165035. doi: 10.3389/fpubh.2023.1165035 (PMC10244627; doi:10.3389/fpubh.2023.1165035)
Supplement: Supplementary file 1 [file Data_Sheet_1.PDF]

ATTITUDES OF VETERINARY STUDENTS TOWARD ANTIBIOTICS

Dear Sir/Madam,
The following questionnaire is a key stage of a sociological research process carried out jointly by the University of Warmia and Mazury, the National Institute of Medicine and the University of Warsaw, with the aim of finding out veterinary students' attitudes toward antibiotics.
Thank you for choosing to participate in this study.
Any response from you is greatly appreciated.
Unless otherwise noted next to the question, please mark only one answer of your choice. The survey is anonymous.
If you give informed consent to participate in the survey, please proceed to the first question. If you do not agree to participate in the survey, please close the form.

METRICS

A. Gender

☐ woman ☐ man ☐ don't want to answer

B. Year of study

☐ I year ☐ II year ☐ III year ☐ IV year ☐ V year ☐ VI year

C. Place of origin/origin

☐ village with a farm
☐ village without a farm
☐ small city with a population of up to 50,000
☐ Medium-sized city with a population of more than 50,000 to 200,000.
☐ large city with a population ofmore than 200,000

D. Professional interests in the context of future work

☐ companion animals
☐ livestock
☐ pharmaceutical industry
☐ scientific work/laboratory work
☐ state administration
☐ other, what...
☐ I haven't decided yet

E. Do you own a pet?

☐ yes
☐ not

I. GENERAL KNOWLEDGE

1. When was the last time you took an antibiotic?

a. ☐ in the last 12 months
b. ☐ 1-2 years ago
c. ☐ more than 2 years ago
d. ☐ never *(go to question 6)*

2. Where did you get the antibiotic you used/used last time?

a. ☐ It was prescribed to me by my family doctor
b. ☐ it was prescribed to me by a doctor of another specialty *(what* specialty*?)* ....
c. ☐ obtained an antibiotic from a veterinarian
d. ☐ It was prescribed to me by my dentist
e. ☐ It was prescribed to me by a nurse
f. ☐ bought/purchased at a pharmacy without a prescription
g. ☐ I had/have an antibiotic at home from a previous treatment
h. ☐ I got/got an antibiotic from a family member/friend
i. ☐ other *(what?)* ....

3. **For what ailments have you recently taken an antibiotic?** *(you can mark more than one answer*)
    a. ☐ cold
    a. ☐ cough
    b. ☐ sore throat
    c. ☐ toothache
    d. ☐ diarrhea

    e. ☐ flu
    f. ☐ acute bronchitis
    g. ☐ pneumonia
    h. ☐ urinary tract infection
    i. ☐ otitis
    j. ☐ Lyme disease

    k. ☐ Shielding e.g. before surgery
    l. ☐ other *(what?)* ....
    m. ☐ I don't remember

4. **Did you use all of the antibiotic, as recommended in terms of dose and duration of intake?**
    a. ☐ yes *(please go to question 6)*
    b. ☐ not

5. **For what reason did you not use the antibiotic as prescribed?**
    a. ☐ I'm still in therapy
    b. ☐ The doctor/dentist advised me to stop the therapy
    c. ☐ I felt/feel better
    d. ☐ Due to side effects (diarrhea, rash)
    e. ☐ antibiotic was not effective
    f. ☐ I forgot/have forgotten
    g. ☐ I wanted/needed to save some for later
    h. ☐ I had/have had the urge to drink alcohol
    i. ☐ Due to other medical reasons *(what?)* ....
    j. ☐ other non-medical reasons *(what?)* ....

6. **Have you encountered a situation in which a veterinarian writes a prescription for an antibiotic for personal use (ad usum prioprium)?**
    a. ☐ yes
    b. ☐ not

7. **As a veterinarian, would you write yourself a prescription for an antibiotic for personal use (ad usum prioprium*?***
    a. ☐ yes
    b. ☐ not

8. **How would you rate your knowledge of antibiotics?** *(Please indicate your answer on a scale where 1 means "very bad" and 6 means "very good"*)

    ☐1 ☐2 ☐3 ☐4 ☐5 ☐6

9. **How would you rate Poles' knowledge of antibiotics?** *(Please indicate your answer on a scale where 1 means "very bad" and 6 means "very good"*)

    ☐1 ☐2 ☐3 ☐4 ☐5 ☐6

10. **Were you taught during your studies about the growing problem of antibiotic resistance?**
    a. ☐ yes
    b. ☐ not

11. **Have your veterinary college classes influenced you to gain more knowledge about the use of antibiotics?** *(Please indicate your answer on a scale where 1 means "definitely not" and 6 means "definitely yes")*

    a. in humans
    ☐1 ☐2 ☐3 ☐4 ☐5 ☐6

    b. in animals
    ☐1 ☐2 ☐3 ☐4 ☐5 ☐6

12. **Did your knowledge of antibiotics gained in your veterinary studies influence the negation of the therapy ordered:**

    a. by a doctor in case of your illness
    a. ☐ yes
    b. ☐ not

    b. by a doctor in case of illness of someone from your family/friends
    a. ☐ yes
    b. ☐ not

    c. by a veterinarian in case of illness of your or your friends' pet
    a. ☐ yes
    b. ☐ not

13. **Below are several views on antibiotics. Please indicate on a scale how much you agree or disagree with each of them** *(where 1 means "strongly disagree" and 6 means "strongly agree")*

    a. antibiotics are effective against viruses

    ☐1 ☐2 ☐3 ☐4 ☐5 ☐6

    b. antibiotics are effective against bacteria

    ☐1 ☐2 ☐3 ☐4 ☐5 ☐6

    c. improper use of antibiotics can cause microorganisms to become resistant to them

    ☐1 ☐2 ☐3 ☐4 ☐5 ☐6

    d. The use of antibiotics will make a person resistant to them

    ☐1 ☐2 ☐3 ☐4 ☐5 ☐6

    e. The use of antibiotics often causes side effects (e.g., diarrhea, headaches, abdominal pain, allergies)

    ☐1 ☐2 ☐3 ☐4 ☐5 ☐6

    f. Antibiotics are effective for the common cold

    ☐1 ☐2 ☐3 ☐4 ☐5 ☐6

    g. Antibiotics are effective for influenza

    ☐1 ☐2 ☐3 ☐4 ☐5 ☐6

h. Doctors often write off antibiotics without needing them

☐1 ☐2 ☐3 ☐4 ☐5 ☐6

i. Bacteria pass information about antibiotic resistance to each other

☐1 ☐2 ☐3 ☐4 ☐5 ☐6

14. Would you like to expand your knowledge regarding the use of antibiotics in animals?

a. ☐ definitely yes
b. ☐ likely yes
c. ☐ rather not
d. ☐ definitely not

15. If your doctor recommends that you take an antibiotic, then:

a. ☐ I would have started/started the treatment fully trusting the doctor
b. ☐ before I would start/start the treatment, I would check/verify the antibiotic information and recommendations in some other source
c. ☐ I would mainly trust myself and my knowledge of the drug

16. If your veterinarian recommends an antibiotic for your pet, then:

a. ☐ I don't have a pet
b. ☐ I would have started/started the treatment fully trusting the vet
c. ☐ before I would start/start the treatment, I would check/verify the antibiotic information and recommendations in some other source
d. ☐ I would mainly trust myself and my knowledge of the drug

II AWARENESS OF THE FUNCTIONING OF PROGRAMS PROMOTING ANTIBIOTIC AWARENESS

1. To what extent - among the sources of information listed below - would you seek information on the use of an antibiotic if it was your pet or the pet of someone close to you? *(Please mark your answer on a scale, where 1 means "to a negligible extent" and 6 means "to a significant extent")*

a. With a veterinarian

☐1 ☐2 ☐3 ☐4 ☐5 ☐6

b. in professional literature on the Internet, electronically

☐1 ☐2 ☐3 ☐4 ☐5 ☐6

c. in professional literature (in hard copy in addition to the Internet)

☐1 ☐2 ☐3 ☐4 ☐5 ☐6

d. in the drug leaflet

☐1 ☐2 ☐3 ☐4 ☐5 ☐6

2. In your opinion, is the issue of antibiotic resistance of microorganisms a significant problem?

a. ☐ yes
b. ☐ Currently not, but will become a problem in the future *(please go to question 4)*
c. ☐ no *(please go to question 4)*

3. If yes, please indicate at what level do you see the problem of antibiotic resistance?

a. ☐ at the regional level
b. ☐ at the national level
c. ☐ at the EU level
d. ☐ globally
e. ☐ don't know, hard to say

4. **Have you heard about the National Antibiotic Protection Program being conducted in Poland?**
   a. ☐ yes
   b. ☐ not

5. **Have you heard about the European Antibiotic Awareness Day?**
   a. ☐ yes
   b. ☐ not

6. **Have you heard of the "One Health" approach?**
   c. ☐ yes
   d. ☐ not

7. **To what extent, in your opinion, do the attitudes/behaviors/advice listed below have an impact on the rise of antibiotic resistance?** *(Please mark your answer on a scale where 1 means "negligible impact" and 6 means "significant impact")*

   a. Overuse of antibiotics in patients/prescription of antibiotics by doctors

      ☐1 ☐2 ☐3 ☐4 ☐5 ☐6

   b. Overuse of antibiotics in patients/prescription of antibiotics by dentists

      ☐1 ☐2 ☐3 ☐4 ☐5 ☐6

   c. misuse of antibiotics in medicine

      ☐1 ☐2 ☐3 ☐4 ☐5 ☐6

   d. misuse of antibiotics in veterinary medicine

      ☐1 ☐2 ☐3 ☐4 ☐5 ☐6

   e. Low awareness of the risks of antibiotic resistance phenomenon

      ☐1 ☐2 ☐3 ☐4 ☐5 ☐6

   f. limited access to microbiological diagnostics

      ☐1 ☐2 ☐3 ☐4 ☐5 ☐6

   g. Use of antibiotics in fattening livestock

      ☐1 ☐2 ☐3 ☐4 ☐5 ☐6

   h. Too long antibiotic therapy in animals

      ☐1 ☐2 ☐3 ☐4 ☐5 ☐6

   i. Using too low doses of antibiotic in animals

      ☐1 ☐2 ☐3 ☐4 ☐5 ☐6

   j. low level of hygiene in breeding

      ☐1 ☐2 ☐3 ☐4 ☐5 ☐6

8. **In your opinion, are there currently recommendations in veterinary medicine for the use of antibiotics in specific clinical situations for specific animal species?** *(Please indicate your answer on a scale where 1 means "definitely no" and 6 means "definitely yes")*

   ☐1 ☐2 ☐3 ☐4 ☐5 ☐6 ☐ don't know

9. **Do you think the public is adequately informed about the following veterinary aspects of ensuring public health, e.g. *Salmonella testing,* meat testing for antibiotics, official controls** *on* **compliance with the withdrawal periods for antibiotics used** *(please indicate your answer on a scale where 1 means "definitely not" and 6 means "definitely yes")*

☐1 ☐2 ☐3 ☐4 ☐5 ☐6

IV KNOWLEDGE OF RESISTANCE TRANSMISSION AND ALTERNATIVE TREATMENTS

1. **In your opinion, should a microbiological test plus an antibiogram be ordered before every antibiotic prescription in humans?** *(Please indicate your answer on a scale where 1 means "strongly disagree" and 6 means "strongly agree")*

   ☐1 ☐2 ☐3 ☐4 ☐5 ☐6 ☐don't know

2. **In your opinion, should selected antibiotics of last resort (e.g., carbapenems) be restricted for use in humans only?**

   ☐1 ☐2 ☐3 ☐4 ☐5 ☐6 ☐don't know

3. **In your opinion, should a microbiological test plus an antibiogram be ordered before any antibiotic prescription in animals?** *(Please indicate your answer on a scale where 1 means "strongly disagree" and 6 means "strongly agree")*

   a. **Before any antibiotic prescription in companion animals**
   ☐1 ☐2 ☐3 ☐4 ☐5 ☐6

   b. **Before any antibiotic prescription in farm animals**
   ☐1 ☐2 ☐3 ☐4 ☐5 ☐6

   c. **After any therapeutic failure following antibiotic administration in companion animals**
   ☐1 ☐2 ☐3 ☐4 ☐5 ☐6

   d. **After any therapeutic failure after antibiotic administration in farm animals**
   ☐1 ☐2 ☐3 ☐4 ☐5 ☐6

4. **You base the answers indicated in the previous question mainly on:**
   a. ☐ knowledge acquired during studies
   b. ☐ Practical experience (acquired in the course of clinical practice - internships, volunteers and others).

5. **If you consider any of the following to be contributing to the likelihood of a population of resistant microorganisms in humans, please indicate to what extent you consider this to be a real threat** *(please* **indicate your** *answer on a scale where 0 means "no impact at all," 1 means "negligible impact," and 6 means "significant impact"***)**

   a. direct contact with companion animals
   ☐1 ☐2 ☐3 ☐4 ☐5 ☐6

   b. Direct contact with farm animals
   ☐1 ☐2 ☐3 ☐4 ☐5 ☐6

   c. Through animal products (eggs, milk, meat)
   ☐1 ☐2 ☐3 ☐4 ☐5 ☐6

   d. through plant-based products
   ☐1 ☐2 ☐3 ☐4 ☐5 ☐6

6. **To what extent do you think the following factors contribute to the increase in the number of multi-resistant strains in livestock** *(please mark your answer on a scale where 0 means "no impact at all," 1 means "negligible impact," and 6 means "significant impact")*

   a. Availability of antibiotic use
   ☐ 0 ☐1 ☐2 ☐3 ☐4 ☐5 ☐6

b. treatment scheme (collective treatment instead of individual treatment)
☐ 0 ☐1 ☐2 ☐3 ☐4 ☐5 ☐6

c. Failure to continue treatment for reasons beyond the doctor's control
☐ 0 ☐1 ☐2 ☐3 ☐4 ☐5 ☐6

d. use of antibiotic growth promoters
☐ 0 ☐1 ☐2 ☐3 ☐4 ☐5 ☐6

e. Cross-contamination during the slaughter process
☐ 0 ☐1 ☐2 ☐3 ☐4 ☐5 ☐6

f. contamination of products of animal origin
☐ 0 ☐1 ☐2 ☐3 ☐4 ☐5 ☐6

g as a result of poor production hygiene
☐ 0 ☐1 ☐2 ☐3 ☐4 ☐5 ☐6

7. **To what extent do you think the following factors contribute to the increase in the number of multiresistant strains in companion animals (***please mark your answer on a scale where 0 means "no impact at all," 1 means "negligible impact," and 6 means "significant impact"*)

a. Availability of antibiotic use
☐ 0 ☐1 ☐2 ☐3 ☐4 ☐5 ☐6

b. treatment scheme (collective treatment instead of individual treatment)
☐ 0 ☐1 ☐2 ☐3 ☐4 ☐5 ☐6

c. the wrong antibiotic
☐ 0 ☐1 ☐2 ☐3 ☐4 ☐5 ☐6

d. Failure to continue treatment for reasons beyond the doctor's control
☐ 0 ☐1 ☐2 ☐3 ☐4 ☐5 ☐6

e. Transfer of resistant bacteria from humans to animals
☐ 0 ☐1 ☐2 ☐3 ☐4 ☐5 ☐6

8. **Are you familiar with alternative treatment regimens to antibiotics and preparations used for therapeutic and preventive purposes of infectious diseases in livestock?**
a. ☐ yes
b. ☐ not

9. **If so, please list:**
....
....

10. **Are you familiar with alternative treatment regimens to antibiotics and preparations used for therapeutic and prophylactic infectious diseases in companion animals?**
a. ☐ yes
b. ☐ not

11. **If so, please list:**
....
....

12. **To what extent, in your opinion, alternative treatment regimens to antibiotics and preparations used for therapeutic and prophylactic purposes of infectious diseases in animals contribute /can contribute to reducing the degree of drug resistance buildup in microorganisms** (please mark your answer on a scale where *0 means "no impact at all,"* 1 means "negligible impact," and 6 means "significant impact")

    ☐ 0 ☐1 ☐2 ☐3 ☐4 ☐5 ☐6
